# Synthesis and evaluation of wound healing properties of hydro-diab hydrogel loaded with green-synthetized AGNPS: in vitro and in ex vivo studies

**DOI:** 10.1007/s13346-022-01121-w

**Published:** 2022-03-31

**Authors:** Mariarosa Ruffo, Ortensia Ilaria Parisi, Marco Dattilo, Francesco Patitucci, Rocco Malivindi, Vincenzo Pezzi, Tzanko Tzanov, Francesco Puoci

**Affiliations:** 1 Department of Pharmacy, Health and Nutritional Sciences, University of Calabria, 87036 Rende, CS Italy; 2 Macrofarm S.R.L, Department of Pharmacy, Health and Nutritional Sciences, University of Calabria, 87036 Rende, Italy; 3 Molecular and Industrial Biotechnology Group, Department of Chemical Engineering, Polytechnic University of Catalonia, Terrassa, Spain

**Keywords:** Diabetic foot ulcerations (DFUs), Silver nanoparticles, Green-synthesis, Hydrogel, Wound healing

## Abstract

In diabetic patients, the presence of neuropathy, peripheral vascular diseases and ischemia, leads to the formation of foot ulcerations with a higher risk of infection because the normal response to bacterial infection is missing. In the aim to control and treat diabetic foot ulcerations (DFUs), wound dressings that are able to absorb exudate, to prevent infections, and to promote wound healing are needed. For this reason, the aim of the present research was to synthetize a biocompatible hydrogel (called HyDrO-DiAb) composed of carboxymethylcellulose loaded with silver nanoparticles (AgNPs) for the treatment of diabetic foot ulcers. In this study, AgNPs were obtained by a green synthesis and, then, were dissolved in a CMC hydrogel that, after a freeze drying process, becomes a flexible and porous structure. The in vitro and in ex vivo wound healing activity of the obtained HyDrO-DiAb hydrogel was evaluated.

**Graphical abstract:**

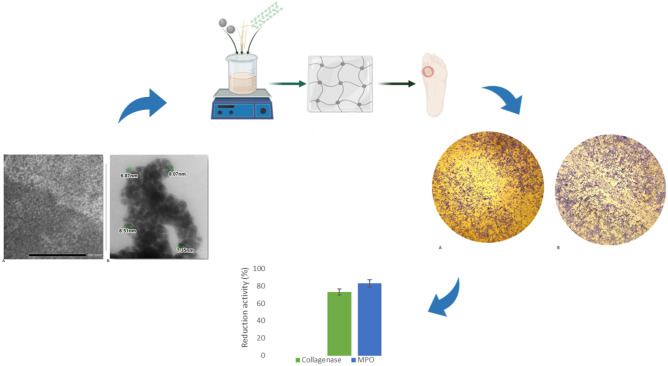

## Introduction

Chronic high blood glucose levels present in diabetic patients increase the risk of developing complications such as heart disease, stroke, blindness, kidney disease, and foot complications [1]. Furthermore, diabetic foot ulcer (DFU), is one of the most severe and difficult complications of diabetes, and it is associated with neuropathy and ischemia [2]. In most cases, healing of DFUs is a slow process, and, for this reason, the presence of infection and heavy exudate causes gangrene and amputation [3]. Among the factors that cause resistance to wound healing, the dysfunction of resident cells represents one of the major causes. Cells like keratinocytes, fibroblasts, macrophages, and lymphocytes are important because they are able to regulate different stages of wound healing, allow the eradication of bacteria, and facilitate the formation of epithelial tissue [4]. In the aim to manage chronic wounds like DFUs, wound dressings should be able to absorb exudate, protect wounds from bacterial infection, and support regeneration and repair of epithelial tissue by providing a suitable environment [5]. Furthermore, to avoid trauma on the wound site, an ideal wound dressing should be non-adherent and easy to apply and remove. For this purpose, hydrogels, which are non-toxic and non-adherent, represent an ideal material for wound healing [6]. The ability to contain high levels of water and the well-known swelling capacity allows the hydrogel to maintain a moist environment around the wound and, at the same time, to absorb exudate from the wound site [7]. Carboxymethylcellulose (CMC) is well known for its water absorbing ability, its swelling capacity, and its biocompatibility [8]. Due to these advantages, CMC-based biomaterials (nanocomposites, films and hydrogels) are widely employed for wound dressing and drug delivery applications [9]. In addition to chronic wound healing (diabetic foot ulcers), CMC has a wide range of acute wound healing applications, including abrasions, first and second-degree burns [10]. Fabrication of bioactive wound dressing materials is significant in wound healing applications because they should have the ability to release biomolecules (such as antibiotics and growth hormones) to the wound site. Hydrogel loaded with silver nanoparticles (AgNPs) represents a promising strategy in the treatment of chronic wounds like DFUs.

AgNPs are well known as antimicrobial agents thanks to their antibacterial, antiviral, and antifungal activities [11]. The ability of silver nanoparticles to cause the death of Gram-positive, Gram-negative, and antibiotic-resistant bacteria makes them a valid broad-spectrum antibacterial agent [12]. Furthermore, AgNPs’ low cytotoxicity and small size allow them to damage microbial cell membranes and cause loss of activity of enzymes, RNA, and DNA, resulting in bacterial death [13]. Furthermore, AgNPs are non-toxic and have high mechanical qualities. They are designed to produce a moist environment surrounding the wound and are capable of promoting continuous oxygen during the wound healing process, and the AgNPs present in the material help to prevent microorganism growth and infection around the wound site [14].

It seems evident that in the last years a great interest about silver and, in particular AgNPs and their applications for wound healing purpose, has been growing and, nowadays, there are well-established knowledge about the antimicrobial properties of silver, that finds application in topical preparations, such as creams and ointments to apply on wounds [15]. Finally, AgNP-based biomaterials are biocompatible, simple to apply and remove from the wound site, inexpensive, due to the availability of materials from natural sources, and present broad-spectrum resistance to many pathogens.

AgNPs were synthetized using a variety of techniques, including physical, chemical, and green synthesis [16]. The last one is the preferred one because, compared to physical and chemical methods, it is less expensive and more ecofriendly [17] and allows the production of nanoparticles from natural extracts [18].

In this study, olive leaves dry extract and *Camellia sinensis* leaves dry extract were used to obtain AgNPs that were loaded into hydrogel composed by CMC to use in the treatment of chronic wounds like DFUs. The obtained nanoparticles were characterized by UV–vis absorption spectroscopy, dynamic light scattering (DLS) and transmission electron microscopy (TEM). Once dimensions and shapes were evaluated, their phenolic content was determined. The obtained nanoparticles were loaded into a CMC-based hydrogel, and the resulting hydrogel (HyDrO-DiAb) was studied. In the aim to evaluate the possible use of HyDrO-DiAb as a wound dressing in the treatment of DFUs, its swelling ability, water retention capacity, anti-inflammatory, antimicrobial, and antioxidant activity were evaluated. Furthermore, the in vitro ability to close a wound and in ex vivo ability to inhibit deleterious wound enzymes were studied.

## Materials and methods 

### Materials

Reagents were purchased from Sigma-Aldrich (Milan, Italy). *Camellia sinensis* leaf dry extract and olive leaves’ dry extract were supplied by Macrofarm srl. The EPISKIN™ RHE/L/13 human skin equivalent kit was obtained from SkinEthic Laboratories (Lyon, France). All solvents were reagents or HPLC grade. EnzChek Gelatinase/Collagenase Assay Kit was provided by Life Technologies (Spain). To in vitro evaluate if HyDrO-DiAb is a skin sensitizer, THP-1 human monocytic leukemia cell line from ATCC was used. Cells were kept in RPMI containing 10% fetal bovine serum (FBS), 0.05 mM 2-mercaptoethanol, 100 units/ml penicillin, and 100 µg/ml streptomycin.

3T3-L1 cells were purchased from ATCC (CL-173) and incubated at 37 °C with 5% CO_2_ in Dulbecco’s modified eagle medium (DMEM), 10% bovine calf serum (BCS), and 1% penicillin/streptomycin.

### Methods

#### Preparation of olive leaves and *Camellia sinensis* extract

In the aim to obtain an aqueous extract by using olive leaves dry extract and *Camellia sinensis* dry extract, the amount of 2.5 g of olive leaves and 1.25 g of *Camellia sinensis* dry extract were dissolved in 100 ml of distilled water at 50 °C and under stirring for 30 min. At the end of extraction time, the obtained aqueous extract (Cs-OLE extract) was filtered by using the Whatman No. 1 filter paper and stored at 4 °C for further analysis.

#### Characterization of the obtained extract

According to the literature [19], the phenolic content of Cs-OLE extract was evaluated by using the Folin-Ciocalteu method. Two milliliters of the obtained extract was mixed with 2 ml of Folin-Ciocalteu reagent and 2 ml of sodium carbonate solution (7.5% w/v). The obtained mix reaction was shaken and incubated for 2 h at room temperature. At the end of 2 h, the absorbance of Cs-OLE extract was measured at 760 nm. The same experimental conditions were performed on a control sample consisting of 2 ml of distilled water, 2 ml of Folin-Ciocalteu reagent, and 2 ml of sodium carbonate solution. The amount of total phenolic compounds was expressed as mg of gallic acid equivalent per g of extract (mgGAE/g). The equation obtained from the calibration curve of gallic acid was used to quantify the amount of total phenolic compounds present in Cs-OLE extract.

#### Green-synthesis and purification of AgNPs

To synthesize silver nanoparticles (AgNPs), 20 ml of Cs-OLE extract was mixed with a solution of AgNO_3_ 0.01 M in a pH-reaction mixture of 8 [20]. The solution was mixed for 20 min at room temperature and, at the end of this time, the nanoparticle solution was centrifuged at 10,000 rpm for 30 min at room temperature. The collected pellets were washed three times and then freeze-dried.

#### Characterization of the obtained AgNPs

To monitor the surface plasmon resonance, the obtained AgNPs were characterized by using Thermo Scientific Evolution 201 UV–vis in the spectral window of 200–800 nm. To evaluate the surface potential and hydrodynamic size, the obtained AgNPs were first diluted to a 1:5 ratio with distiller water and then, sonicated. The obtained AgNP suspension was analyzed by using a zeta sizer Particles Size Analyzer 90 Plus (Brookhaven Instrument Corporation, New York, NY, USA). Finally, the morphology and distribution of synthetized AgNPs were further examined with high resolution TEM (JEM-1409Plus).

The phenolic content of AgNPs was evaluated by using Folin-Ciocalteu method [19]. Ten milligrams of the obtained nanoparticles was mixed with 2 ml of distilled water, 2 ml of Folin-Ciocalteu reagent, and 2 ml of sodium carbonate solution (7.5% w/v). After 2 h of incubation at room temperature, the absorbance was measured at 760 nm, and the obtained data were expressed as mg of gallic acid equivalent per g of nanoparticles (mgGAE/g).

#### Preparation and characterization of HyDrO-DiAb

For the preparation of hydrogel (HyDrO-DiAb), CMC was dissolved in water in a concentration of 2% w/v and left under stirring for 30 min at room temperature. After the dissolution of CMC, AgNPs (0.01% w/w) were dissolved in the solution and maintained at room temperature for 6 h under stirring. After that, citric acid, as a crosslinking agent, was added and the obtained mix reaction was incubated for a further 6 h at room temperature. To obtain the final hydrogel loaded with AgNPs, the prepared solution was freeze-dried and then, stored at room temperature for further analysis.

To evaluate the swelling ability of HyDrO-DiAb, 30 mg of tested sample was placed into a tared 5-ml-sintered glass filter (Ø 10 mm; porosity, G3), weighed, and left to swell in an alkaline PBS (pH 8) to mimic chronic wound conditions [21]. The filter was left in PBS for 24 h and, after that, the excess of the solution was removed by percolation, centrifugated at 2000 rpm for 10 min and, finally, weighed. The filter tare was measured after centrifugation with only water. The water content percentage (WR%) was calculated at different time intervals according to Eq. (1):

1 $$WR \;\% = \frac{W{s}-W{d}}{W{d}} \times 100$$

where *W*_s_ was weight of swollen hydrogel, while *W*_d_ was weight of dried hydrogel. Each experiment was carried out three times.

Once swollen, the hydrogel was used to evaluate water retention capacity. The swollen sample was incubated at 37 °C with 70–75% relative humidity and water retention capacity was measured using Eq. (2):

2 $$Rw= \frac{W{t}}{W0 }\times 100$$

where *W*_t_ is weight of sample at different time intervals, and *W*_0_ is weight of sample before incubation.

To in vitro evaluate the release of AgNPs from the tested sample, the amount of 50 mg of HyDrO-DiAb was immersed in 6 ml of PBS (0.1 M, pH 8) and placed in a dialysis bag (12,000–14,000 Da MWCO), which was sealed at each end with clamps [22].

The dialysis tube was immersed in a flask containing 40 ml of the same PBS and incubated for 8 h at 37 °C ± 0.5 °C in constant agitation in a water bath.

The amount of 1 ml of sample was withdrawn and, at selected time intervals of 1, 2, 4, 6, and 8 h, was replaced by the same volume of fresh PBS. The amount of released AgNPs was quantified spectrophotometrically at 430 nm, by using the equation obtained from the calibration curve of AgNPs. All the experiments were carried out in triplicates.

#### Cell viability assay

Cell viability studies were assessed using MTT assay on 3T3 L1 cell line. In the performed study, the reduction of yellow 3-(4, 5-dimethyl thiazol-2-yl)-2, 5-diphenyl tetrazolium bromide (MTT) by mitochondrial succinate dehydrogenase, which can only occur in metabolically active cells, [23] was evaluated. Briefly, cells were seeded in 96-well plates at 1 × 10^4^ cells/well and maintained at 37 °C with 5% CO_2_ in DMEM containing 10% bovine calf serum (BCS) and 1% penicillin/streptomycin. Then, the medium was removed and replaced with a fresh one containing different concentrations of HyDrO-DiAb (45–100 μg/ml). After 24 h of incubation, cell viability was determined by adding 10 μl of MTT solution (1 mg/ml) to each well. After 1 h of incubation at 37 °C in an atmosphere containing 5% CO_2_, 100 μl of DMSO for formazan crystal solubilization, was added. After 10 min of incubation in dark conditions, the absorbance of reduced MTT was evaluated at 570 nm in a microplate reader (Synergy h1 Hybrid reader Biotek). Cell viability was expressed as a percentage compared to control wells.

#### Wound healing scratch assay

The wound healing ability of HyDrO-DiAb was assessed by wound healing scratch assay [24]. 3T3-L1 cells were seeded at a density of 5 × 10^5^ cells/well in 6-well plates and incubated for 24 h in an incubator at 37 °C in a CO_2_ (5%) atmosphere. Wound healing scratch assay was performed by using a p200 pipet trip which scraped the cell monolayer, creating a “scratch” [24]. The debris was removed by washing cells with PBS, which was then replaced with 2 ml of culture medium containing 100 μg/ml of HyDrO-DiAb. Wells in which hydrogel was not incubated were used as a control. After 24 h of incubation, the cells were stained with Coomassie blue brilliant, and the scratched area was visualized with a microscope (Olympus ckx53) under phase-contrast optics (× 4 magnification). The percentage of wound closure was calculated using the Image J software [25] and expressed as reported in Eq. (3):

3 $${Wound\; clousure}\; (\%)= \frac{{A}_{t}-{A}_{0}}{{A}_{t}}\times 100$$

where *A*_0_ is the area of the wound measured immediately after scratching, and *A*_*t*_ is the area of the wound measured after 24 h.

##### MIC

The evaluation of minimum inhibitory concentration (MIC) (the lowest concentration of the formulation at which the organisms do not demonstrate visible growth) of HyDrO-DiAb was determined against *Staphylococcus aureus* (ATCC6538), *Escherichia coli* (ATCC8739) and *Pseudomonas aeruginosa* (ATCC9027). In each sterile tube, different concentrations of tested sample (from 0.1 to 50 μg/ml) were added to TSB (Tryptone Soy Broth) and to 100 μl of adjusted bacterial solution (CFU ~ 10^7^). Turbidity in the tested tubes indicated the growth of microorganisms and, so, MIC is considered the lowest concentration of hydrogel that causes color change [26].

#### In vitro antioxidant activity

The antioxidant activity of the HyDrO-DiAb was tested using DPPH ((2,2-diphenyl-1-picryl-hydrazyl-hydrate) and ABTS (2,2′-azino-bis (3-ethylbenzothiazoline-6-sulfonic acid) radical scavenging assays [27]. For the DPPH radical scavenging assay, 30 mg of tested hydrogel was added to 1 ml of distilled water and to 4 ml of an ethanolic solution of the enzyme (200 μM). Finally, in the aim to obtain a final volume of 10 ml, ethanol was added to the mix reaction. The tested sample was incubated at room temperature, in the dark, for 15 min. At the end of this time, the residual DPPH concentration was measured at 517 nm against a control prepared in the same reaction conditions but without hydrogel. The radical scavenging capacity was calculated according to Eq. (4).

The ABTS radical scavenging activity of hydrogel was evaluated by following the literature with some modifications [28]. The amount of 30 mg of tested sample was added to 2 ml of ABTS solution and the resulting mix reaction was incubated for 6 min in the dark, under stirring, and at room temperature. At the end of 6 min, the absorbance was measured at 734 nm, and the radical scavenging activity was measured according to Eq. (4). A control sample was prepared in the same experimental conditions but without hydrogel.

#### In vitro anti-inflammatory activity

The anti-inflammatory estimation of HyDrO-DiAb was studied by evaluating the ability of tested sample to inhibit BSA denaturation, by using BSA as a model protein [29]. One hundred milligrams of tested hydrogel was suspended in 2 ml of a PBS pH 7.4 solution of BSA (1 mM). In order to cause the denaturation of BSA, the mixture was incubated for 30 min at 37 °C. The absorbance of the reaction was read at 660 nm and the inhibition of BSA denaturation was estimated using Eq. (4).

4 $$Inhibition\;(\%)=\left(\frac{A_0-\;A_1}{A_0}\right)\times 100$$

where *A*_0_ is the absorbance of control sample and *A*_1_ is the absorbance of HyDrO-DiAb.

#### In ex vivo ability to inhibit wound enzyme*s*

Wound exudates, which were extracted using the UrgoClean dressing (from Urgo Medical) of a patient with a foot ulcer (Hospital de Terrassa (Spain), were used to perform in ex vivo studies. To obtain wound exudates, 1 g of UrgoClean dressing, which was used as dressing in a patient with diabetic foot ulcer, was soaked in 5 ml of PBS pH 7.4 for 10 min [30]. Thereafter, the supernatant was vortexed for 10 min at 10,000 rpm at 4 °C and finally stored in the fridge at 4 °C for further use.

To evaluate the ability of hydrogel to inhibit in ex vivo myeloperoxidase (MPO) activity, the amount of taurine chloramine produced by the MPO/H_2_O_2_/Cl^−^ system, was evaluated. Hypochlorous acid can be trapped with taurine to form a stable chloramine, which can then be detected to reveal enzyme activity. Taurine chloramine is normally assayed by measuring the bleaching of 3,3′,5,5′-tetramethylbenzidine. To do this, 2 mg of HyDrO-DiAb was mixed with 750 μl of PBS (50-mM pH 6.5 with 200 mM of NaCl, and 6.67 mM of taurine), 100 μl of H_2_O_2_ (1 mM), and with 150 μl of wound fluid diluted 10 times. The enzymatic reaction was incubated for 30 min at 37 °C and then stopped by adding 25 μl (1 mg/ml) of catalase solution. To evaluate the amount of produced taurine chloramine at 650 nm, 50 μl of the detection reagent (2 mM 3,3′,5,5′’-tetramethylbenzidine in 10% DMSO, 100 ml NaI in 400-mM acetate buffer pH 5.4) was added to the enzymatic reaction. The obtained results were expressed as a percentage of MPO inhibition and were compared to those obtained in a control sample prepared without hydrogel [31].

Fluorescently labeled gelatin substrate ((EnzChek kit, Thermo Fisher Scientific) is digested by collagenase, which causes the release of a fluorescent peptide that increases the fluorescence of the supernatant. In the presence of an inhibitor of collagenase, gelatin is not digested and, consequently, there is a decrease in the fluorescence value of the supernatant.

The ability of HyDrO-DiAb to inhibit collagenase was performed by incubating 30 mg of sample with 400 μl of wound fluid, previously diluted 2 times.

The mix reaction was left for 24 h at 37 °C and, at the end of this time, 100 μl of incubated sample was transferred to a 96-well plate and was mixed with 80 μl of buffer. Twenty microliters of gelatin substrate (250 μg/ml) was added to each well, and the change of fluorescence was monitored at excitation/emission 493/528 nm. A sample without hydrogel was used as a control and the collagenase activity in it was considered as 100% [31].

#### In vitro skin sensitization (h-CLAT) OECD 442E

The purpose of the test human cell line activation test (h-CLAT) is to evaluate the sensitizing potential of HyDrO-DiAb in accordance with the method described in the Organization for Economic Cooperation and Development (OECD) 442E [32] and in the 158 EURL-ECVAM protocol (European Union Reference Laboratory for alternatives to animal testing). In this study, a monocyte cell line, named THP-1, was used as a prototypic blood-derived immunologically active cell. On these cells, the expression of two co-stimulatory molecules, CD54 and CD86, was tested and 2,4-dinitrochlorobenzene (DNCB), a well-known contact sensitizing agent, was used as a positive control. An increased expression of CD54 and CD86 on monocytes is a signal of activation of the immune response, derived from the exposition of potentially sensitizing contact allergens. In the performed test, human monocytic leukemia cell line named THP-1 (ATCC TIB-202) was kept in RPMI containing 10% FBS, 0.05-mM 2-mercaptoethanol, 100 units/ml penicillin, and 100 µg/ml streptomycin. To evaluate their reactivity, THP-1 cells were exposed to DNCB and nickel sulfate, which were used as positive controls, and to lactic acid, which was used as a negative control. Once their reactivity was evaluated, cells were seeded in a 96-well flat-bottom plate at a density of 1.6 × 10^5^ cells/well and, after 24 h, the culture medium was mixed 1:1 with the tested hydrogel and controls. The culture medium was used as a negative control. At the end of incubation time, the cells were centrifugated and re-suspended in a flow cytometry staining buffer (FACS Buffer) which contained iodure propidium (PI) for cytometry analysis. The concentration that caused 25% of cell mortality (CV75 value) was calculated and used as the highest concentration in the final test.

Once the CV75 value was determined, HyDrO-DiAb was solubilized in phosphate buffer at a concentration equal to 100-fold the 1.2 CV75. Then, to obtain different stock solutions ranging from 0.335 × CV75 to 1.2 × CV75, 1:1.2 serial dilutions are made. The obtained stock solutions were diluted 1:50 into the culture medium and, finally, a further 1:2 dilution factor was used for the h-CLAT test. In the cited test, the culture medium and DNCB (4 μg/ml) were used as negative and positive controls, respectively. Cells were exposed to the tested sample for 24 h at 37 °C with 5% CO_2_ and, at the end of incubation time, they were centrifugated, resuspended in FACS buffer, and divided into three aliquots. Three aliquots of cells were, furthermore, centrifugated, resuspended in FACS buffer containing 0.01% of gamma globulins and incubated at 4 °C for 15 min. At the end of the incubation time, the cells were centrifugated and incubated with fluoresceinated anti-CD86, anti-CD54 or mouse IgG1 (control isotype) antibodies for 30 min at 4 °C.

Finally, the cells were washed with FACS buffer and resuspended in the same buffer in the presence of a PI solution. Cell viability and the expression levels of CD86 and CD54 were analyzed with flow cytometry. Cell viability was calculated using the following Eq. (5):

5 $${Cell\; viability\; (\%) =\; ( }\frac{{Living\; cells}}{{Acquired\; cells}}{)}\times 100$$

The concentration that showed 75% of THP-1 cell survival (CV75) was calculated by using the following Eq. (6)

6 $${Log}\; CV75 = \frac{(75-B) \times {Log}\; (C) - (75-A) \times {Log}\; (D)}{A-B}$$

where *A* is cell viability > 75%; *B* is cell viability < 75%; and *C* or *D* denotes the concentration corresponding to cell viability *A* or *B*. The obtained CV75 value was used to define the highest concentration of tested hydrogel that could be used for the measurement of CD86/CD54 expression in the final test. After the treatment of cells with the tested sample, the expression of CD86 and CD54 was analyzed by flow cytometry. The relative fluorescence intensity (RFI) of CD86 and CD54 for positive control and hydrogel-treated cells, respectively, was calculated according to the following Eq. (7), where MFI is the mean fluorescence intensity that is proportional to the expression of co-stimulatory molecules:

7 $${RFI}\;=\frac{ {MFI}\; {of}\; {sample}\; {with}\; {cells}- {MFI}\; {of}\; {sample}\; {with}\; {isotype}\; {cells}\;}{ {MFI}\; {of}\; {solvent}\; {with}\; {cells}- {MFI}\; {of}\; {solvent}\; {with}\; {isotype}\; {cells}}$$

#### In vitro skin irritation OECD 439

To predict skin irritation from chemicals, which cause a decrease in cell viability, the reconstructed human epidermis test method (OECD 439) was used [33]. The EpiDerm™ reconstructed human epidermal (RhE) plate was equilibrated overnight in a humidified incubator at 37 °C and 5% CO_2_. Then, 25 mg of HyDrO-DiAb was applied directly to the top of the epidermis surface, and its effect was compared to that obtained from sodium dodecyl sulfate SDS (5%) and PBS, which were used as positive and negative controls, respectively. After 60 min of contact between RhE and the tested sample, the tissues were rinsed with PBS, transferred into 2 ml of fresh medium, and incubated for 42 h. All the tests were performed thrice. At the end of incubation time, tissue viability was assessed by MTT reduction measurement. To do this, RhE tissues were transferred into a 24-well plate in which MTT medium (1 mg/ml) was added. The tested plate was placed in a humidified incubator (37 °C, 5% CO_2_) for 3 h and, at the end of this time, the RhE tissues were removed from the MTT medium. To extract formazan salt, the tissues were transferred to a 24 well-plate containing 2 ml of isopropanol and then shaken at 120 rpm at room temperature for 2 h. The amount of 200 μl of each extraction solution was transferred into a 96-well plate and the optical density (OD) of the extracted formazan was determined at 570 nm using a microplate reader (Synergy h1 Hybrid reader Biotek). Isopropanol was used as a blank vehicle. Cell viability was expressed as a percentage and calculated using the following Eq. (8).

8 $${Viability \; (\%)} = \frac{(OD \; sample\; \times\; 100)}{OD \; negative \; control}$$

#### Statistical analysis

The obtained data were expressed as the mean ± standard deviation (SD) and analyzed using one-way analysis of variance. *p* values < 0.05 were considered statistically significant.

## Results and discussion

### Preparation and characterization of the extract

Among the plant extracts which could be used for green synthesis of AgNPs, *Camellia sinensis* and olive leaves extract can be considered a perfect mix, thanks to their polyphenolic composition.

*Camellia sinensis* leaves’ dry extract is rich in polyphenolic compounds such as catechins, which represent 70–80% of total polyphenols [34]. Catechins present in tea leaves are characterized by polymeric and monomeric catechins, condensed tannins, proanthocyanidins, and catechin derivatives [35]. Considering the total catechin content, 50–80% is represented by epigallocatechin-3-gallate (EGCG), while the remaining part is represented by other catechins such as epicatechin-3-gallate (ECG), epigallocatechin (EGC), and epicatechin (EC). The other important components of green tea are flavonols, which contribute to the antioxidant property of *Camellia sinensis* leaves [36].

Olive leaves from Calabrian *Olea europaea* L., the other plant chosen for green synthesis of AgNPs, present a high concentration of phenolic compounds and several types of flavonoids which are well known to have antioxidant properties [37]. The phenolic compounds present in olive leaves are classified into acids, flavonoids, and secoiridoids. The latter are the components present in higher concentrations and, among these, Oleuropein is the first secoiridoid isolated from olive leaves, followed by its derivatives such as hydroxytyrosol and tyrosol.

*Camellia sinensis* leaves and olive leaves’ dry extracts were used to obtain an aqueous extract (Cs-OLE extract) with a high concentration of phenolic compounds able to reduce Ag^+^ in Ag^0^ (31).

The phenolic content of Cs-OLE extract, used to synthesize AgNPs, was measured by the Folin–Ciocalteu method, and the obtained results evidence a concentration of phenolic compounds of 15 ± 0.5 mg GAE/g of extract.

### Green synthesis and characterization of AgNPs

**Fig. 1:**
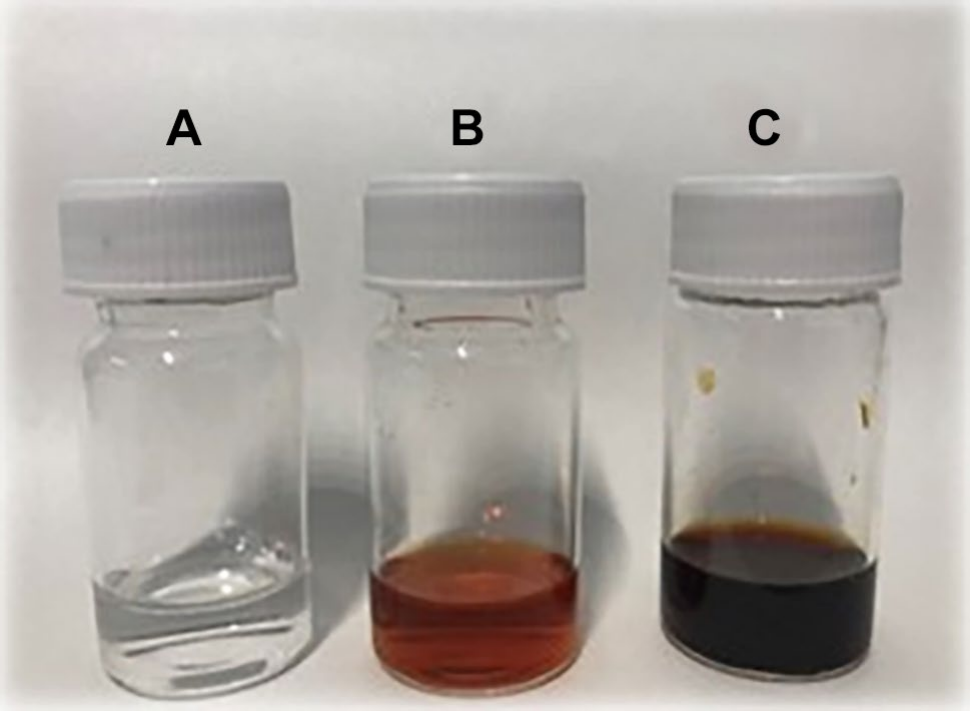
AgNO_3_ 0.01 M **(A)**, Cs-OLE extract **(B)**, AgNPs **(C)**

In the present study, AgNPs were synthesized by the reduction of silver ions in the presence of Cs-OLE extract characterized by a high concentration of phenolic compounds. Indeed, thanks to their well-known antioxidant activity, phenolic compounds could be used to reduce silver ions (Ag^+^) to metallic ions (Ag^0^). To do this, 20 ml of the extract was mixed with a 0.01 M AgNO_3_ solution in a pH 8 reaction mixture for 20 min at room temperature. At the end of reaction time, the formation of AgNPs was confirmed by the formation of a yellowish-brown color (Fig. 1) caused by excitation of surface plasmon resonance (SPR) [38].

**Fig. 2:**
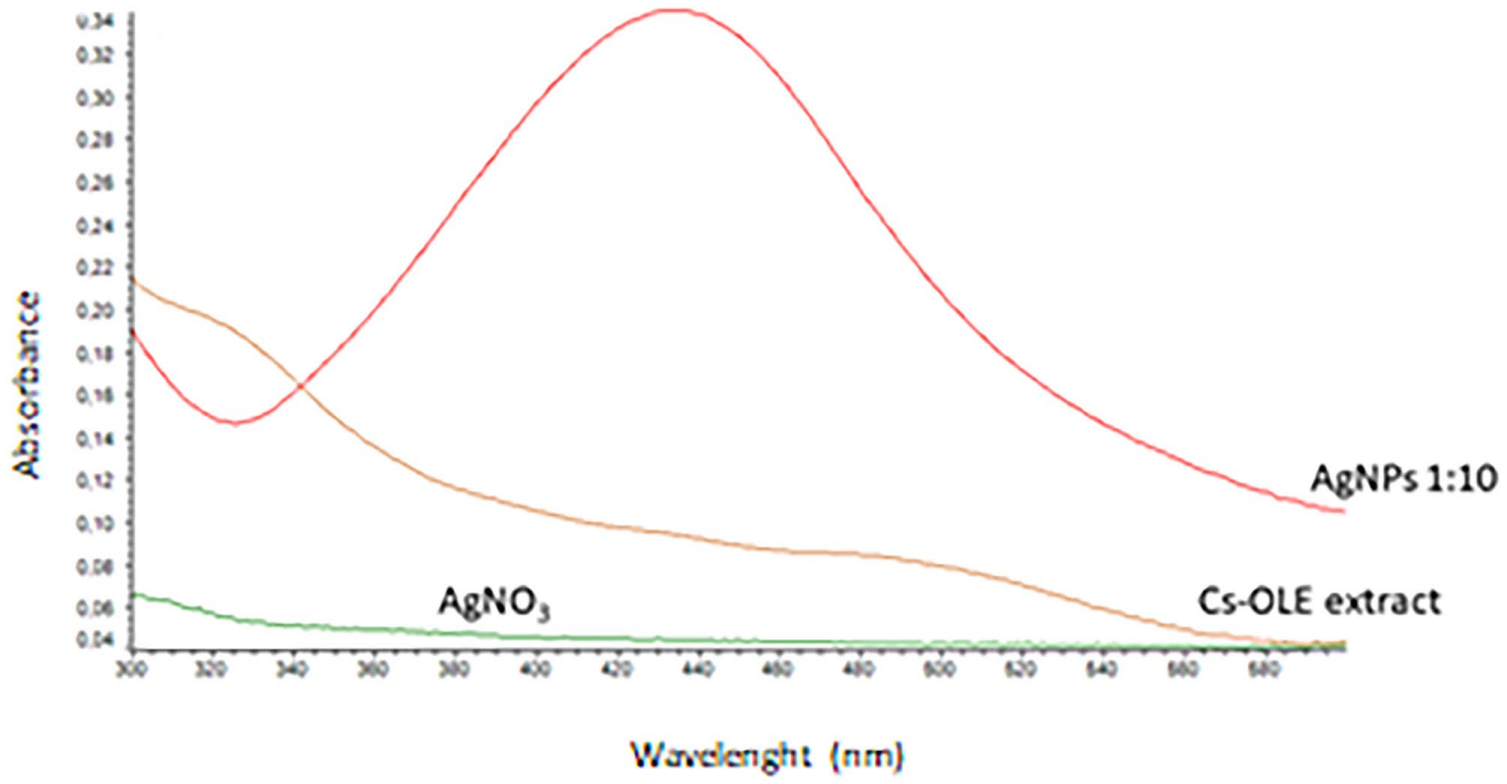
UV–vis absorption spectra of AgNO_3_, Cs-OLE extract and synthetized AgNPs (diluted in a ratio 1:10 with distilled water**)**

AgNPs, in fact, present a particular optical phenomenon (SPR) due to their conduction electrons on the metal surface, which undergo a collective oscillation when they are excited by light in a specific wavelength [39]. Size, shape, and type of synthetized nanoparticles influence SPR band which appears in the range of 400–500 nm in UV–vis spectroscopy [40]. In this study, the SPR band of green-synthetized AgNPs was obtained around 430 nm (Fig. 2) with an absorbance of 0.34 after a dilution of 1:10 with water. Previous studies suggested that UV–visible absorption spectrum of AgNPs is sensitive to their formation and that the value of absorbance is influenced by particle diameter and shape [41] and, in particular, nanoparticles with small diameters present a larger absorption value.

**Fig. 3:**
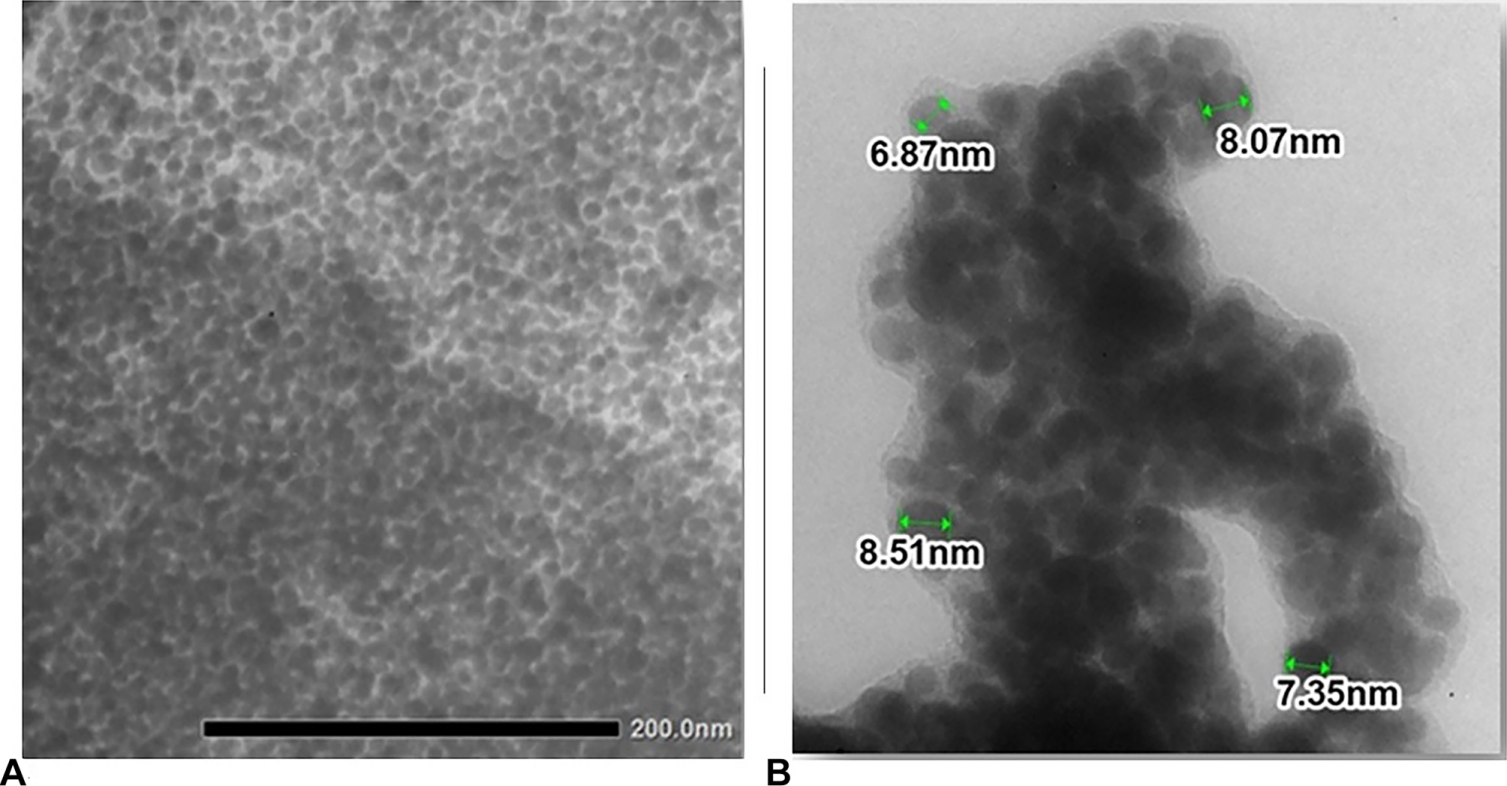
Morphologies of AgNPs **(A)** and dimension observed in TEM images **(B)**

In the aim to evaluate size, shape, and dimension of nanoparticles, TEM and DLS instruments were used. TEM images (Fig. 3) showed monodisperse AgNPs with a spherical shape and without the phenomenon of agglomeration, which is characteristic of AgNPs synthetized with a plant extract [42] that prevents aggregation of particles. The obtained AgNPs showed a spherical shape (Fig. 3A) with a dimension between 6.87 and 8.51 nm (Fig. 3B).

The hydrodynamic size of synthetized AgNPs was measured in solution and the obtained results (Table 1) showed that the measured size was different from that measured with TEM. This is probably due to the hydration layers on the surface of AgNPs. DLS results confirmed that the obtained nanoparticles were monodisperse with a polydispersity index of 0.179.

**Table 1:** Dynamic light scattering measurements of AgNPs

**Sample**	**Mean diameter (nm)**	**Polydispersity index**
AgNPs	88 ± 1.6	0.179 ± 0.02

To evaluate the phenolic content of AgNPs and, so, the amount of phenolic compounds that did not react with AgNO_3_ in the reduction reaction, the Folin-Ciocalteu method was performed. The obtained results showed that the amount of 1.71 ± 0.02 mg equivalent of gallic acid is present in 1 g of nanoparticles and, so, phenolic compounds of Cs-OLE extract do not react completely with AgNO_3_ and confer antioxidant activity to the nanoparticles as will be demonstrated in the further results.

### Swelling ability, water retention capacity, and studies release of HyDrO-DiAb

Hydrogels based on CMC could be considered a promising material for chronic wound therapy, since they exhibit important swelling properties which make them a suitable biomimetic environment for cells and tissues. Good swelling properties make hydrogels a perfect medium for sustained and slow release of various therapeutic agents to wound sites such as AgNPs and, at the same time, hydrogels should absorb exudate and maintain a moist wound environment [43]. To evaluate HyDrO-DiAb as a dressing in the treatment of DFUs, in vitro swelling behavior and moisture retention capacity of the prepared hydrogel were evaluated.

The results were compared to those obtained in a hydrogel made entirely of CMC.

**Fig. 4:**
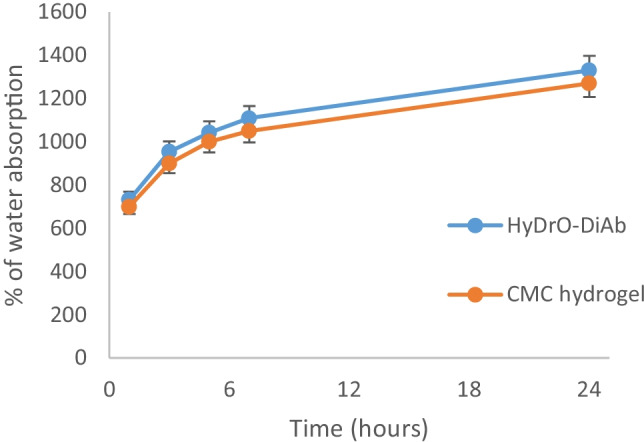
Water absorption capacity of HyDrO-DiAb and CMC hydrogel. All the results are reported as mean values ± SD (*n* = 3)

The obtained results (Fig. 4) showed a fast rate of absorption in the first 6 h for both HyDrO-DiAb, and the hydrogel composed only of CMC. These findings show that the water holding capacity of the tested hydrogels is much greater than their weight and that the addition of AgNPs to the CMC hydrogel has no effect on the biopolymer’s hydrophilic nature.

**Fig. 5:**
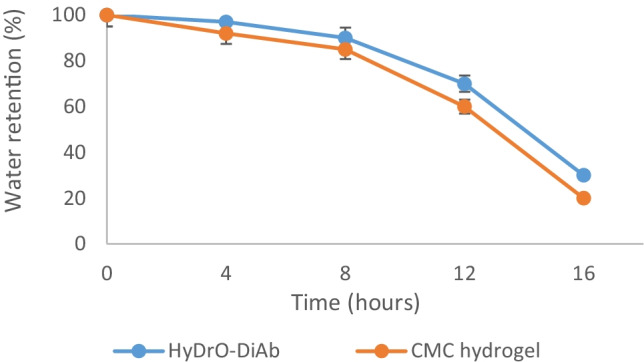
Water retention capacity of HyDrO-DiAb and CMC hydrogel. All the results are reported as mean values ± SD (*n* = 3)

The ability of wound dressing to retain moisture is an important feature to evaluate because epithelial migration and autolytic debridement occur in the presence of optimal moisture content. Both HyDrO-DiAb and hydrogel composed only of CMC showed a fast decrease in moisture content (Rh) with a total loss of water of 70–80% after 24 h (Fig. 5). The obtained results evidenced the ability of the prepared hydrogel to retain moisture and, so, its possible use as a wound dressing.

**Fig. 6:**
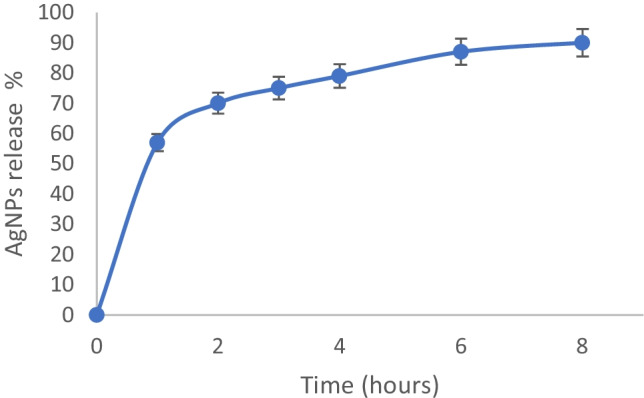
In vitro study release of AgNPs from hydrogel. All the results are reported as mean values ± SD (*n* = 3)

The high-water absorption capacity of the tested hydrogel makes it a perfect medium for sustained and slow release of AgNPs to wound sites and, for this reason, the amount of released AgNPs from hydrogel was tested at different times. The obtained results (Fig. 6) showed that nanoparticles were released in a slow and sustained manner for 8 h and, so, this hydrogel can work as a carrier of AgNPs.

In the first 2 h, there was a burst release of AgNPs with 71.5 ± 0.5% of total released AgNPs followed by a slow release with 90 ± 0.1% of released nanoparticles.

### Cell viability assay

**Fig. 7:**
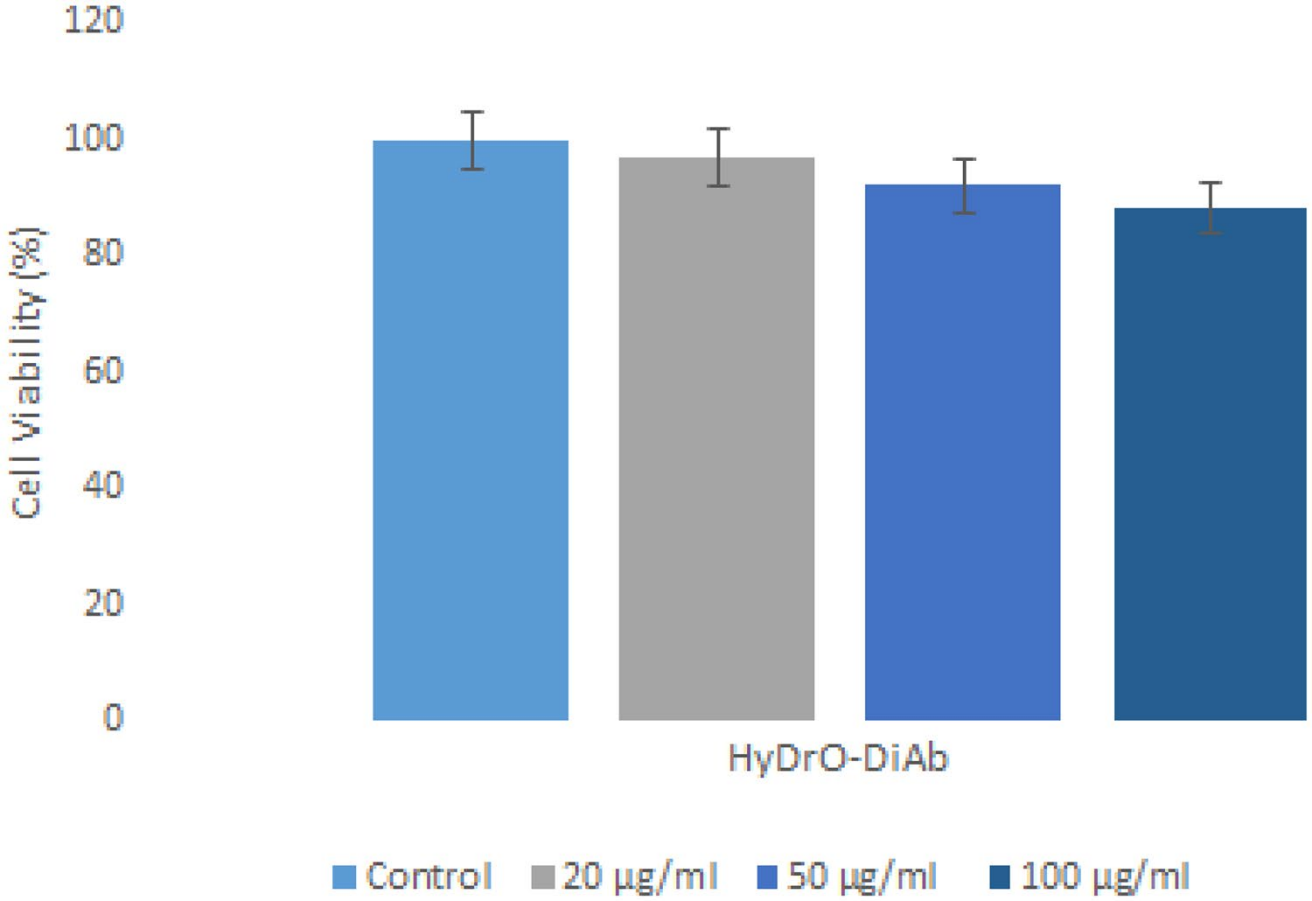
Viability of 3T3 fibroblasts cells after 24 h of incubation at 37 °C with HyDrO-DiAb. All the results are reported as mean values ± SD (*n* = 3)

The evaluation of the cytotoxicity of a hydrogel that should be used as a dressing for chronic and infected wounds is an important consideration. The biocompatibility of hydrogel was evaluated by using 3T3 fibroblast cells because they act as producers of growth factors, which control cell growth and differentiation. For this reason, it is very important to minimize the loss of function of fibroblasts by using a biocompatible material [44]. The obtained results indicated that hydrogel exhibited low toxicity to cell fibroblasts. 3T3 fibroblast cells showed 88–97% of viability after 24 h of incubation with tested hydrogel. Since the decrease in fibroblast viability for the tested sample did not reach 20%, it is possible to confirm that the prepared hydrogel presented a good biocompatibility profile for chronic wound treatment. Figure 7 presents the results of toxicity tests of HyDrO-DiAb, which was tested at different concentrations.

### Wound healing scratch assay

**Fig. 8:**
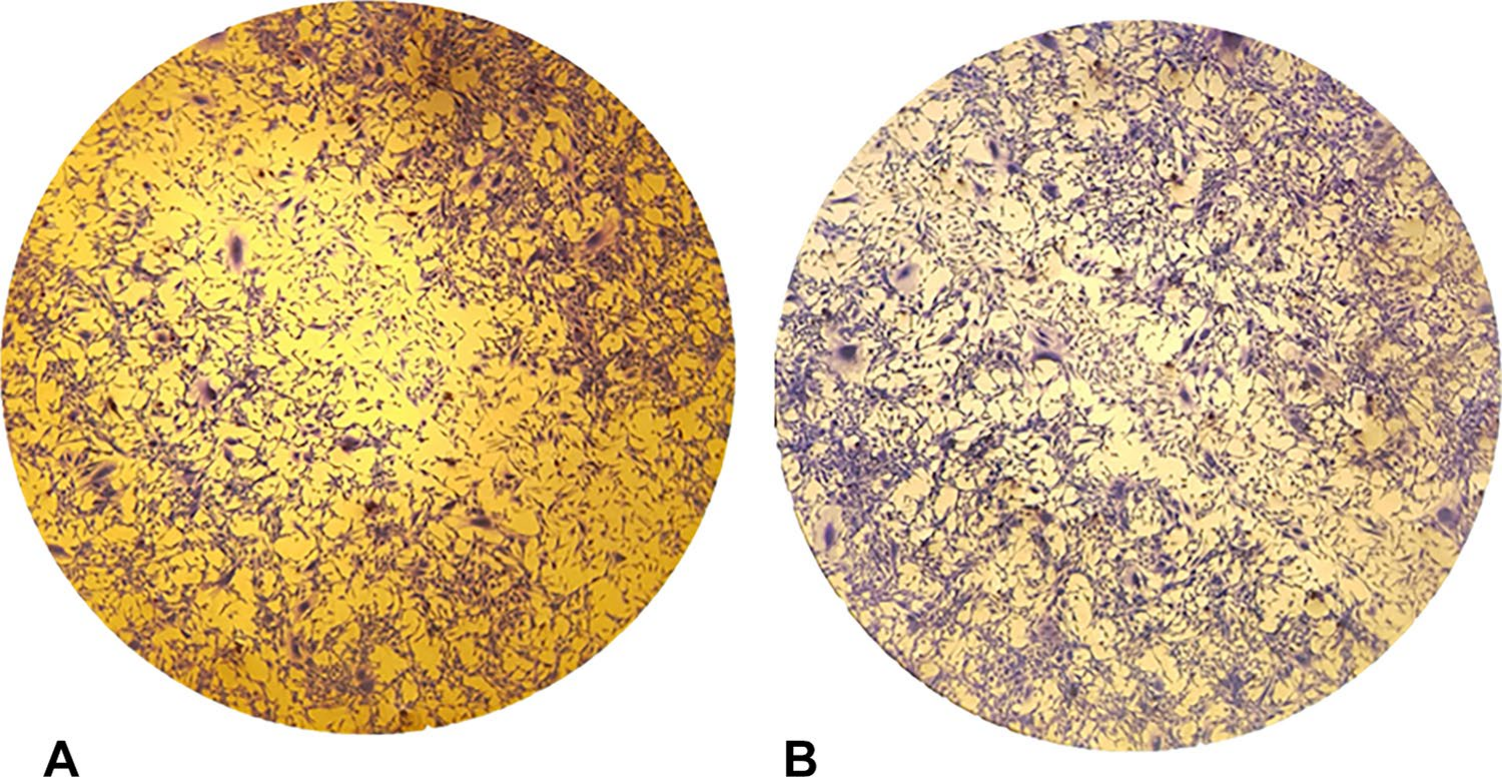
Wound healing activity of HyDrO-DiAb on 3T3 fibroblast cells by scratch method **(A)** and evaluated after 24 h **(B)**

Wound healing is characterized by several phases such as homeostasis, inflammation, proliferation, and remodeling which occur, thanks to the activity of several cell types such as keratinocytes, fibroblasts, and progenitor cells [24]. The cellular and molecular events that involve the wound healing process are chemokines, growth factors, interleukins, and cytokines that allow interactions between cells and between cells and extracellular matrix (ECM) [45]. The in vitro wound healing activity of HyDrO-DiAb was performed on 3T3 fibroblast cells. As it can be seen from Fig. 8, after 24 h of treatment of cells with the amount of 100 μg/ml of hydrogel, the wound closure percentage was 75 ± 0.3%.

The obtained results demonstrated that the tested HyDrO-DiAb could have a positive effect on the wound healing process. As already reported, this wound healing process could be ascribable to the antibacterial activity of silver nanoparticles, which are able to modulate cytokine production and to mitigate inflammation processes [46].

### MIC

The in vitro antibacterial activity of HyDrO-DiAb was checked against *S. aureus*, *E. coli*, and *P. aeruginosa* by using the microdilution method, and the obtained results were reported as minimum inhibitory concentration (MIC). HyDrO-DiAb's MIC values against *E. coli*, *S. aureus*, and *P. Aeruginosa* were 5.15 g/ml, 30 g/ml, and 27 g/ml, respectively.

When AgNPs enter into contact with wound fluid of the wound surface, they get oxidized and release Ag^+^ ions which exercise antibacterial activity in three ways: interact with sulfur-containing proteins of the bacterial cell membrane, causing its damage; enter inside the bacteria, causing DNA disruption; attack the bacterial respiratory chain, causing inhibition of cell division and cell death [13].

### In vitro antioxidant and anti-inflammatory activity of HyDrO-DiAb

**Fig. 9:**
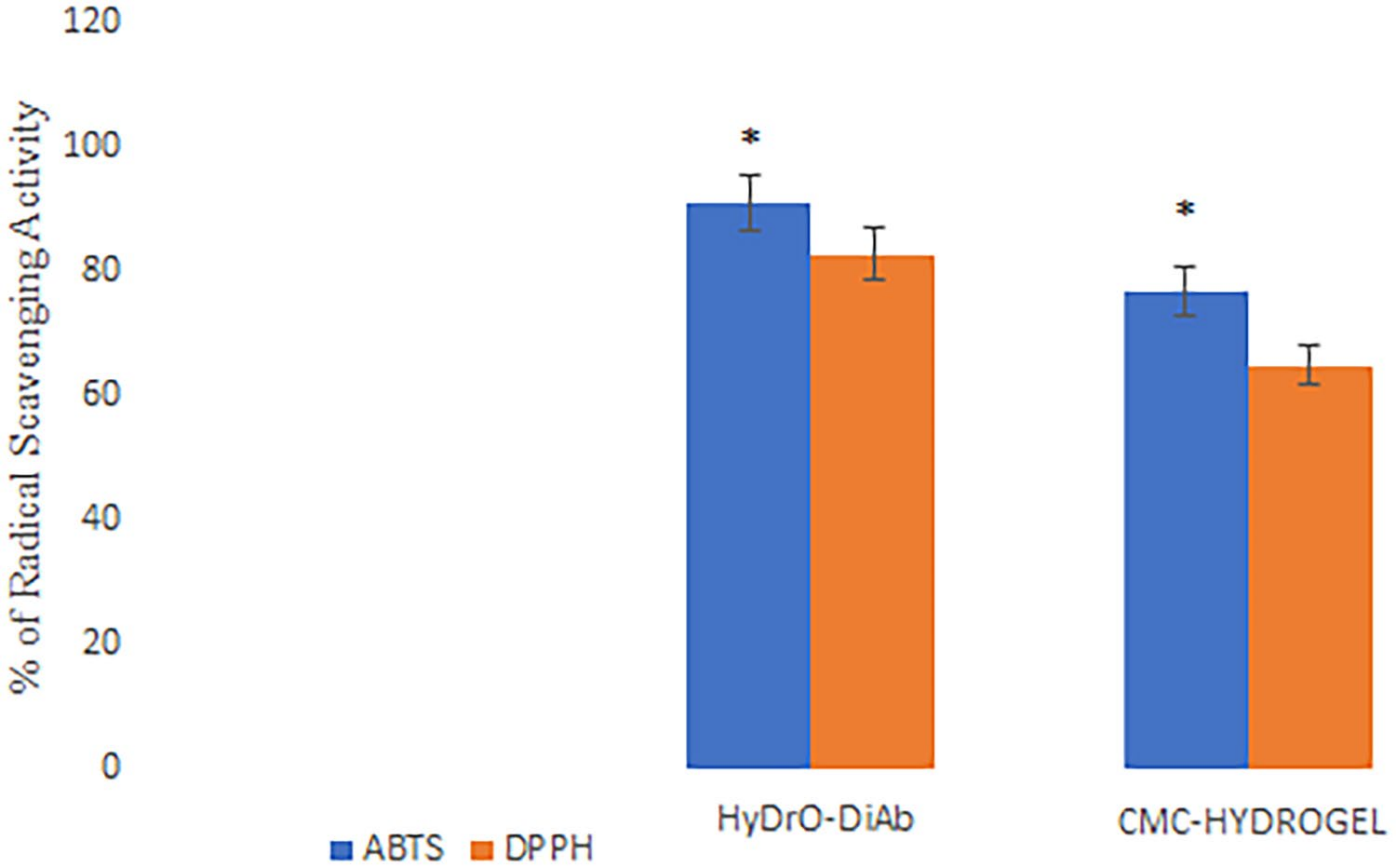
ABTS and DPPH radical scavenging activity of HyDrO-DiAb and CMC-hydrogel. All the results are reported as mean values ± SD (*n* = 3). *p* values < 0.05 were considered statistically significant

The inflammatory phase of chronic wound healing is characterized by the production of oxygen species (ROS) by immune cells which, doing this, provide a defense against microorganisms. However, in a chronic wound, the overexpression of ROS causes damage to the ECM and cells [47] with a consequent delay of wound healing. For this reason, it is very important to reduce ROS. The radical scavenging ability of HyDrO-DiAb was assessed by ABTS and DPPH assays, and the obtained results were compared to those obtained by a hydrogel composed only by CMC. The obtained data evidenced that HyDrO-DiAb shows a radical scavenging activity above 91% and 83% for ABTS and DPPH radical, respectively (Fig. 9). Hydrogel composed only of CMC presents a lower radical scavenging activity (Fig. 9), probably due to the absence of AgNPs. Antioxidant components of Cs-OLE extract, which were used for green synthesis of nanoparticles, probably influenced the reducing capacity of AgNPs and, so, maybe not all phenolic compounds of the extract have been oxidized to form NPs. This hypothesis was confirmed by evaluating the amount of phenolic compounds which remain in nanoparticles after reduction reaction with AgNO_3_ (previously reported).

It is well known that denaturation of tissue proteins, which lose their secondary, tertiary, and quaternary structure, leads to inflammatory disease [48]. So, tissue protein denaturation becomes a marker for the inflammatory state. For this reason, the anti-inflammatory activity of the obtained hydrogel was carried out by estimating its capacity to prevent BSA denaturation. The tested hydrogel showed a good percentage of inhibition (53 ± 0.6%) if compared with that manifested by CMC hydrogel (27 ± 0.3%). The obtained results confirmed the anti-inflammatory activity of HyDrO-DiAb, which, with its antibacterial activity, becomes a potential dressing that could be used for the treatment of inflammatory state presents in chronic wound sites like DFUs.

### In ex vivo ability to inhibit wound enzymes

In the aim to evaluate the ability of HyDrO-DiAb to improve the wound healing process, its ability to inhibit MPO and collagenase was tested. Indeed, these enzymes are considered a potential therapeutic target because they are present in high levels in chronic wound sites.

In the first host immune defense of humans, polymorphonuclear neutrophils are activated and produce MPO which is able to generate hypochlorous acid (HClO) and to neutralize invading microorganisms [30]. HClO is the most potent bactericidal agent produced in humans that, if it is produced in higher quantities, can oxidize different biomolecules and inhibit the wound healing process [49]. Furthermore, the inhibition of metalloproteinases, such as collagenase, is an important factor in wound repair because, if they are present in the exudate, they cause excessive degradation of ECM [50]. Collagenase, indeed, is responsible for the hydrolysis of triple helical regions of collagen and, consequently, causes the loss of activity of this protein which, under physiological conditions, contributes to the reconstruction of damaged tissues. The MPO and collagenase inhibition of HyDrO-DiAb was evaluated in ex vivo by using foot ulcer exudates.

**Fig. 10:**
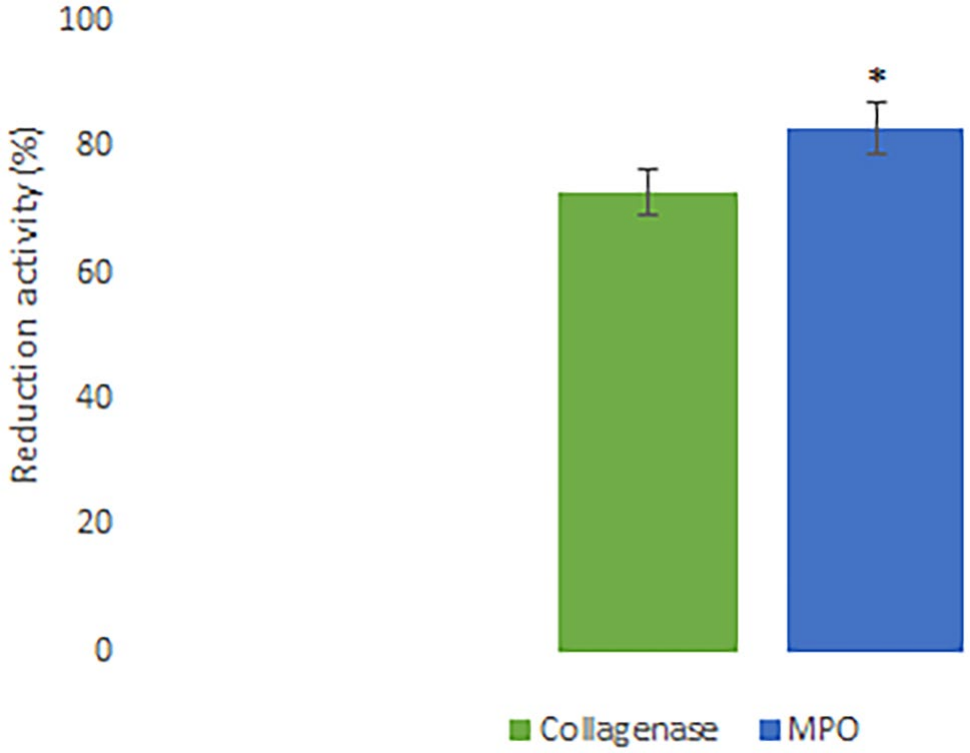
In ex vivo ability of HyDrO-DiAb to inhibit collagenase and MPO activity. All the results are reported as mean values ± SD (*n* = 3). *p* values < 0.05 were considered statistically significant

The obtained results (Fig. 10) showed a residual activity of collagenase of 27 ± 0.5%. These findings suggest that HyDrO-DiAb could be used to treat chronic wounds such as DFUs because it inhibits collagenase activity by 73 ± 0.3%, in ex vivo. This important activity of hydrogel could be related to its ability to absorb wound exudate that, consequently, causes a reduction of collagenase activity. At the same time, the presence of phenolic compounds in AgNPs can induce conformational changes in the secondary structure of collagenase and, so, inhibit its activity. Furthermore, the tested HyDrO-DiAb can reduce MPO activity by 84 ± 0.5% (Fig. 10). Most probably, this strong inhibition activity of the tested hydrogel was due to the burst release of AgNPs in the first 2 h, which evidenced high antioxidant activity and, therefore, a scavenge property against radical and non-radical species such as HClO.

The obtained results confirm that the application of HyDrO-DiAb seems to be a promising approach in wound healing treatment because it is able to reduce MPO and collagenase activity, which are responsible for oxidation of biomolecules and excessive degradation of ECM, respectively.

### Determination of in vitro skin sensitization

**Fig. 11:**
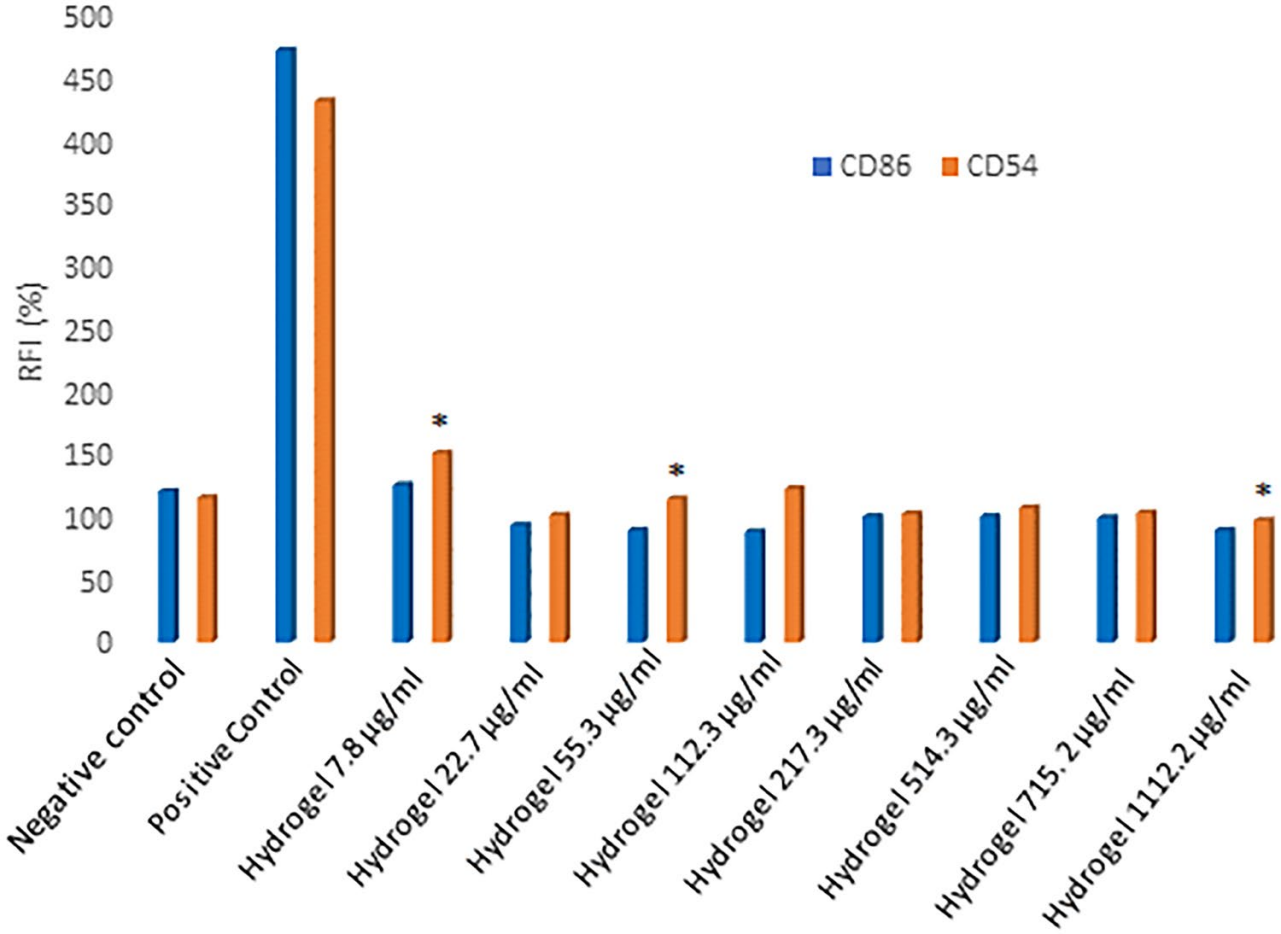
Expression of CD86 and CD54 after treatment with negative control, positive control and HyDrO-DiAb. Different concentrations of HyDrO-DiAb were tested to evaluate if it is a skin sensitizer. All results are reported as mean values ± SD (*n* = 3). *p* values < 0.05 were considered statistically significant

The h-CLAT test was used to evaluate if the tested hydrogel, HyDrO-DiAb, is a skin sensitizer or non-sensitizer. To predict the sensitization of tested sample, RFI% values of CD86 and CD54 were considered. Indeed, if the RFI% value of CD86 is ≥ 150%, and/or if the RFI% value of CD54 is ≥ 200% in at least two independent runs, the sensitization prediction is considered as positive. On the other hand, if the RFI% value of CD86 is < 150% and/or if the RFI% value of CD54 is < 200%, the sensitization prediction is considered as negative. The guideline reports a protocol divided into different steps. After an initial control on the reactivity of THP-1 cells (reactivity check), the dose finding assay is used to define the concentration range to be used for the evaluation of CD54 and CD86 expression levels. THP-1 cells were exposed to test HyDrO-DiAb for 24 h and to evaluate CV75, PI uptake was evaluated by flow cytometry. The obtained results, reported in Fig. 11, confirmed that the tested sample is not a skin sensitizer.

### Determination of in vitro skin irritation

When a test substance is applied to the skin for up to 4 h, it produces a reversible damage to the skin. This substance is considered an irritant to the skin [51]. On the basis of the guidelines of the Organization for Economic Cooperation and Development (OECD TG431 and TG439), MTT (viability) on the RhE model was used to in vitro evaluate if the tested hydrogel was skin irritant or corrosive. The MTT assay was used to evaluate the activity of mitochondrial reductase, which predicts cell viability. In the aim to study skin irritation of the tested sample in an accurate and reliable way, HyDrO-DiAb was added to the apical side of the EpiDerm™ RhE issues. These 3D tissues were largely used because they are highly sensitive. In fact, the permeability of their non-viable stratum corneum is 5–200-fold greater than normal human skin [52]. HyDrO-DiAb was tested in this study and the percentages of EpiDerm™ cell viability, after exposure to sample and controls, were over 50%, indicating that the tested sample was non-irritant, compared with 5% SDS (positive control), which showed strong irritancy.

## Conclusions

The prepared hydrogel loaded with AgNPs (HyDrO-DiAb) provided good swelling ability and water retention capacity, and so, the addition of green-synthetized AgNPs to the hydrogel of CMC does not modify the hydrophilic nature of the biopolymer. Moreover, HyDrO-DiAb can work as a carrier of AgNPs because the obtained release results showed that nanoparticles were released in a slow and sustained manner for 8 h. The Scratch test assay revealed that, after 24 h of treatment of the cells with the amount of 100 μg/ml of hydrogel, the wound closure percentage was 75 ± 0.3%, and so, the obtained hydrogel could affect the wound healing process positively. This important activity was confirmed in ex vivo studies, in which the ability of HyDrO-DiAb to inhibit MPO and collagenase was tested. The obtained data highlighted that HyDrO-DiAb could be used for the treatment of chronic wounds like DFUs because it inhibits collagenase and MPO activity by 73 ± 0.3% and 84 ± 0.5%, respectively. HyDrO-DiAb also presents good antioxidant, anti-inflammatory, and antimicrobial activity, so it could be used for the treatment of inflammation and infection present in DFUs. Finally, to evaluate the safety of the prepared hydrogel, cytotoxicity, skin sensitization, and skin irritation were evaluated. The obtained results confirmed the safety and biocompatibility of HyDrO-DiAb.

## Data Availability

Not applicable.
